# The Physiological Action of Cocaine on the Frog

**Published:** 1885-04

**Authors:** H. W. Biggs

**Affiliations:** Studies made in the Physiological Laboratory at Berlin


### ART. X.—THE PHYSIOLOGICAL ACTION OF COCAINE ON THE FROG (*RANA ESCULENTA*), WITH SPECIAL REFERENCE TO ITS LOCAL ACTION ON ORGANS AND TISSUES.

BY H. W. BIGGS, M.D.

(Studies made in the Physiological Laboratory at Berlin.)

In the following experiments the hydro-chlorate of cocaine, prepared by Werck, of Darmstadt, was used. It is non-crystalized, of a dull white color, has a slightly bitter taste, nearly neutral reaction, and is very soluble in water, alcohol, ether and chloroform. The solutions used were all made with water, and were 1, 2 and 4 per cent. in strength.

*Local Action.*—If the foot of a frog be immersed in a solution of cocaine (2 per cent.) for a few minutes, very marked anæsthesia of the skin is produced, or if a solution (2 or 4 per cent.) is applied to the mucous membrane of the mouth, the same action is observed. When a few drops of a 4 per cent. solution are instilled into the eye of a frog, there is caused after a short time almost complete anæsthesia of the conjunctiva and cornea. The action on the pupil is not constant, but usually a moderate degree of mydriasis is produced.

*General Action.*—The effects of cocaine given by hypodermic injection begin to manifest themselves very quickly, and in a few minutes have reached their maximum. When large doses are used, the action is seen almost immediately, and the duration and severity of the symptoms are in proportion to the amount administered. After small doses the frogs begin to ercover at the end of a few minutes, but if the quantity given is large, it is many hours, and may be several days, before the effects have entirely disappeared. Lethal doses are from .04 grm. upwards, and when death occurs from the effects of cocaine it is only after the lapse of twenty-four or thirty-six hours.

When a small toxical dose of cocaine (.005 grm.) is injected under the skin of a frog, there are usually at first no perceptible effects. In some cases there is a short period of excitement, but this is not always present, nor is it as a rule very strongly marked when present. The frog soon becomes quiet, he moves less quickly after stimulation is applied, and his respirations are somewhat decreased in number. In a few minutes he becomes perfectly motionless, changes his position only after considerable irritation, and then with an evident effort. Presently his head drops, the respirations become slower, and the pupils somewhat dilated. Twenty or thirty-five minutes after receiving the poison, he lies flat on his abdomen and neck with the hind legs extended, and is unable to raise or balance himself. If strong stimulation is applied he makes a vain effort to spring, which sometimes takes the form of a partial clonic convulsion. There is marked loss of voluntary power over his extremities, and this appears earlier and is much greater in the posterior extremities than in the anterior. The action of the heart is somewhat slower than normal. Not long after this he begins to recover; his respirations are more frequent and deeper, he raises his head, and is able to balance himself when not disturbed. The last function to return is complete voluntary power in his limbs. An hour or an hour and a half after receiving the poison the frog has become quite normal again.

After moderate toxical doses given hypodermically, as after small doses, there is sometimes a short period of excitement, but this even when present soon gives way to the depressing action of the drug. The animal becomes perfectly quiet, the respirations slow and superficial, and the heart's action diminished both in force and frequency. At the end of seven or eight minutes the frog can no longer balance himself, and lies flat on his abdomen and neck with the posterior extremities extended as before. His toes being pinched, he makes two or three powerless efforts to move, and then remains quiet. The loss of power and also sensation is more marked, and appears earlier in the hindlegs than in the forelegs, as in the first experiment. The pupils are dilated. After about twenty minutes he becomes perfectly relaxed, and remains in any position in which he is placed. His respirations are imperceptible, and only weak relax movements occur after severe mechanical stimulation, although a moderately strong interrupted current calls forth muscular contractions.

An hour later the frog lies in the same position. Neither any action of the heart nor any respiratory movement is perceptible, and the reflexes are even less marked than before.

About two hours and a half after receiving the poison, the animal begins to show signs of recovery. It makes some slight voluntary movements, and the reflexes are stronger. Three hours later he turns over on his belly, and irritation calls forth contractions of a clonic character. There are as yet no signs of returning respiration. In twelve or fourteen hours the frog has entirely recovered.

When large toxical doses (.025 grm.) of cocaine are given to the frog hypodermically, the effects begin to manifest themselves almost instantly. All the phenomena observed in the previous experiments here follow each other in rapid succession, and in a much more intense form. There is no period of excitation. In two or three minutes respiration is labored, pupils dilated with opisthotonos, heart's action slow, and reflexes greatly diminished. The frog has a very anxious expression, and in a few minutes drops motionless on the table, extended at full length. Severe irritation calls forth only a very weak response. Respiration ceases with the lungs in inspiration. After many hours, if the dose has not been lethal, signs of returning life begin to show themselves, but it is two or three days before the animal entirely recovers. After the exhibition of a lethal dose the heart continues to act for twenty-four or thirty-six hours, and then stops in diastole.

*Action on the Heart.*—When a frog has been poisoned by cocaine given by hypodermic injection, the action of the heart is either unaffected or there is a diminution in the rapidity of its pulsations. After very small doses (0.001 grm.) there is no perceptible influence. When a somewhat larger amount is given (0.005 grm.) the frequency of the heart's action is reduced from eight to twelve pulsations a minute, and it becomes irregular and intermittent. In two or three hours the influence of the drug passes off, and the action of the heart becomes perfectly normal again. After very large toxical doses (0.025 grm.) the number of pulsations may be reduced to six or eight per minute. The heart becomes engorged with blood, and its action is very much labored and irregular. The alternate action of the auricles and the ventricles is replaced by an irregular action, and long pauses occur in the pulsations of the ventricles. The ventricles are affected earlier and more severely than the auricles, and when paralysis finally occurs, if it has been a lethal dose, the ventricles cease to pulsate a long time before the auricles.

EXPERIMENT I.

Medulla destroyed.—Heart exposed.—M. IV. Sol. Cocaine, (1 per cent. equals .0025 gramme) injected hypodermically.

| Time. | Minutes After Cocaine Given. | Pulsations of Ventricle in 15 Seconds. | Pulsations of Auricles in 15 Seconds. | Amount Cocaine. | Remarks. |
|---|---|---|---|---|---|
| 11.20 | | 12.12 | 12.12 | | |
| 11.24 | | 12.12 | 12.12 | .0025 gr. | |
| 11.26 | 2 | 11.11 | 11.11 | | |
| 11.30 | 6 | 11.11 | 11.11 | | Action somewhat labored. |
| 11.40 | 16 | 10.11 | 10.11 | | |
| 11.45 | 21 | 10.11 | 10.11 | | |
| 11.55 | 31 | 10.11 | 10.11 | | |

### EXPERIMENT II.

Medulla destroyed.—Heart exposed.—M. VIII. Sol. Cocaine, (2 per cent. equals 0.01) injected hypodermically.

| Time. | Minutes After Cocaine was Given. | Pulsations of Ventricles in 15 Seconds. | Pulsations of Auricles in 15 Seconds. | Amount Cocaine. | Remarks. |
|---|---|---|---|---|---|
| 3.43 | | 11.12 | 11.12 | | |
| 3.45 | | 12.12 | 12.12 | .01 gr. | |
| 3.47 | 2 | 11.11 | 11.11 | | |
| 3.57 | 12 | 8.8 | 8.8 | | Irregular. Occasional long pauses in action. |
| 4.01 | 16 | 8.8 | 8.8 | | |
| 4.04 | 19 | 8.9 | 8.9 | | |
| 4.10 | 25 | 7.7 | 7.7 | | Irregular and intermittent. Occasional pauses of ventricles, while auricles continue to act. |
| 4.15 | 35 | 7.7 | 8.9 | | |
| 4.30 | 50 | 7.8 | 8.9 | | |
| 4.36 | 56 | 7.8 | 10.9 | | |
| 4.52 | 72 | 8.9 | 9.8 | | |
| 5. | 80 | 9.9 | 9.9 | | Acting regularly. |

### EXPERIMENT III.

Medulla destroyed.—Heart exposed.—M. x. Sol. Cocaine, (4 per cent. equals 0.025 gramme) injected hypodermically.

| Time. | Minutes After Cocaine was Given. | Pulsations of Ventricles in 15 Seconds. | Pulsations of Auricles in 15 Seconds. | Amount Cocaine. | Remarks. |
|---|---|---|---|---|---|
| 4.12 | | 11.12.11 | 11.12.11 | | |
| 4.14 | | 11.12 | 11.12 | 025 gr. | |
| 4.17 | 3 | 4.3.4 | 8.9 | | Very irregular and labored in its action. |
| 4.21 | 7 | | 7.8 | | |
| 4.24 | 10 | 3.3.3 | 6.6 | | Heart engorged with blood. Ventricles cannot empty themselves completely. Very irregular and intermittent. |
| 4.27 | 13 | 3.2.3 | 4.5 | | |
| 4.33 | 19 | 3.2 | 6.6 | | |
| 4.35 | 21 | 3.2.0 | 6.6 | | |
| 4.40 | 26 | 0.0.0 | 4.4 | | Pause of 60 seconds in action of ventricles. |
| 4.50 | 36 | 3.2.0 | 5.5 | | |
| 5 | 46 | 0.0.3 | 5.6 | | |

*Local action on the heart.*—If the heart of a frog be exposed, and three or four minims of a 4 per cent. solution of cocaine be injected directly into the ventricle, the heart is immediately paralyzed and stops in systole. If now, as a control experiment, into the heart of another frog the same quantity of water or normal salt solution be injected, no effect is produced save that arising from the physical injury, and this soon passes away; but afterward the injection of the same quantity of a 4 per cent. solution of cocaine causes as before immediate paralysis of the heart in systole.

Again, when three or four minims of a 4 per cent. solution of cocaine are injected into the median abdominal vein, or into one of the great veins of the heart, paralysis either immediately or after two or three beats results. In this case the heart stops in diastole.

The local application by means of a camel's-hair pencil of a weak solution (1 per cent.) of cocaine to the heart muscle, causes a temporary diminution in the frequency of its pulsations.

EXPERIMENT IV.

Medulla destroyed.—Heart exposed.

| Time. | Pulsations of Heart in One Minute. | Cocaine Applied in 1 Per Cent. Sol. | Remarks. |
|---|---|---|---|
| 10.25 | 33 | | |
| 10.50 | 34 | | |
| 11 | 34 | 1 per cent. Sol. | |
| 11.4 | 24 | | Heart somewhat distended with blood. |
| 11.13 | 29 | 1 per cent. Sol. | |
| 11.18 | 24 | | |
| 11.34 | 34 | 1 per cent. Sol. | |
| 11.36 | 17 | | Action labored and irregular, but soon becomes normal in frequency and character. |
| 11.44 | 30 | 1 per cent. Sol. | |
| 11.47 | 17 | | Irregular. Heart engorged. |
| 12.10 | 26 | | |

### Action of Cocaine in Substance on the Heart Muscle.

EXPERIMENT V.

Medulla destroyed; Heart exposed; Cocaine in substance applied to both auricles and ventricles. In about three minutes the heart had ceased to beat. The ventricles are in systole and the auricles are in diastole.

EXPERIMENT VI.

Conditions same as in V.

Cocaine in substance applied to heart. After about five minutes the heart had ceased to pulsate. The ventricles are in systole, and the auricles are in diastole.

Four or five hours in neither of these cases had the heart begun to pulsate again.

EXPERIMENT VII.

Conditions same as in previous experiments ; results same ; ventricles are firmly contracted and pale; auricles large and distended with venous blood. In the last case, eighteen hours later, the heart was found to be pulsating again slowly and feebly. It continued to act for about six hours and then again stopped.

In a number of succeeding experiments it was observed that in most cases, after many hours, the heart commenced again to act, but this was not true in all cases, and when the cocaine was injected directly into the cavity it never occurred. In all cases the ventricles were in systole, and in all but one the auricles were in diastole.

*Influence on Respiration.*—After small doses of cocaine (.0025 grm.) there is a slight increase in the frequency of the respirations at first, but soon they become much diminished in number, and may be reduced to six or eight per minute. These are very superficial, and sometimes for a short time they entirely disappear.

With medium-sized doses (from .005 grm. to .01 grm.) the temporary rise in the number of respirations per minute is less marked, and the depressing effects of the drug are more quickly seen. The decrease in the frequency is much more rapid than after small doses, and the respiratory movement usually becomes imperceptible in about twenty-five or thirty minutes.

After large toxical doses (from .025 grm., upwards) there is no resemblance of a temporary increase. The respirations rapidly diminish in number and change in character, and usually in ten minutes have become imperceptible. After these large doses respiration stops with a very full inspiration, in other cases apparently after expiration. Respiration does not appear again until the end of at least twenty-four hours, and often it is a much longer time.

### EXPERIMENT VIII.

M. IV. Sol. Cocaine, (1 per cent. equals .0025 gramme) injected hypodermically. Frog free under glass jar.

| Time. | Minutes After Cocaine was Given. | Respirations. in 15 Seconds | Amount Cocaine. | Remarks. |
|---|---|---|---|---|
| 10.15 | | 18 | .0025 gr. | |
| 10.17 | 2 | 20 | | |
| 10.20 | 5 | 24 | | Somewhat irregular, but rather deep. |
| 10.22 | 7 | 20 | | |
| 10.25 | 10 | 17 | | |
| 10.28 | 13 | 14 | | Quick, jerky and superficial. |
| 10.30 | 15 | 12 | | |
| 10.33 | 18 | 10 | | |
| 10.36 | 21 | 7 | | |
| 10.38 | 23 | 5 | | Long. |
| 10.40 | 25 | 8 | | Very superficial. |
| 10.44 | 29 | 16 | | Very superficial, scarcely preceptible. |
| 11.12 | 57 | 12 | | |
| 11.20 | 1 h. 5 m. | 18 | | Natural. |

### EXPERIMENT IX.

M. VIII. Sol. Cocaine, (2 per cent. equals .01 gramme) injected hypodermically. Frog free under glass jar.

| Time. | Minutes After Cocaine was Given. | Respirations in 15 Seconds. | Amount Cocaine | Remarks. |
|---|---|---|---|---|
| 10.48 | | 18 | | |
| 10.50 | | 18 | .01 gr. | |
| 10.53 | 3 | 22 | | |
| 10.55 | 5 | 20 | | |
| 10.57 | 7 | 18 | | |
| 11.03 | 13 | 16 | | |
| 11.07 | 17 | 9 | | Very superficial. |
| 11.15 | 25 | 8 | | Scarcely preceptible. |
| 11.28 | 38 | None | | |

EXPERIMENT X.

M. x. Sol. Cocaine, (4 per cent. equals .025 gramme) injected hypodermically. Frog free under glass jar.

| Time. | Minutes After Cocaine was Given. | Respirations in 15 Seconds. | Amount Cocaine. | Remarks. |
|---|---|---|---|---|
| 11.33 | | 18 | | |
| 11.35 | | 18 | .025 gr. | |
| 11.37 | 2 | 16 | | Difficult respiration. |
| 11.38 | 3 | 14 | | |
| 11.39 | 4 | 12 | | Very superficial. |
| 11.40 | 5 | None | | |

*Action on Nervous System.*—Spinal Cord: when small doses of cocaine have been administered to a frog, the reflexes seem for a short time to be slightly increased. After larger doses the period of heightened reflexes is very short and then follows a period of much longer duration, when they are decidedly diminished. When large toxical doses have been given, there is no period of greater irritability, but almost immediately the general reflexes are very much lessened, and there is soon almost complete loss of all response to mechanical or chemical stimulation. Loss of reflex to strong electrical stimulation never occurs except just before death.

Sensory and Motor Nerves (according to B. von Aurep): "Small doses heighten the irritability of the sensory fibres without having any effect on the motor filiments. Larger doses cause, after a slightly increased irritability of the sensory fibres of short duration, a diminution of the same. Large toxical doses almost completely paralyze the sensory filiment, and diminish greatly the irritability of the motor. Tactile reflexes are much diminished. The pneumogastric nerves are completely paralyzed after doses of moderate size. The small have no perceptible influence on the inhibitory fibres of the pneumogastrics. They either remain after as before the poisoning, responding to the same strength of current, or they are wholly paralyzed."

EXPERIMENT XI. (from B. von Aurep.)

The heart exposed. Both pneumogastric nerves divided.

| Time. | Contractions of Ventricles in 15 Seconds. | Contractions of Auricles in 15 Seconds. | Amount Cocaine. | Remarks. |
|---|---|---|---|---|
| 8.30 | 13.14.14 | 13.14.14 | | |
| 8.40 | 14.14.14 | 14.14.14 | | Stimulation right pneumogastric. |
| | 7.4.2 | 7.4.2 | | |
| 8.55 | 13.14.14 | 13.14.14 | | Stimulation of left pneumogastric nerve. |
| | 3.3.1 | 3.3.1 | | |
| 9.05 | 14.14.14 | 14.14.14 | 0.03 gr. | |
| 9.10 | 7.6.6 | 7.6.6 | | Stimulation of right pneumogastric. |
| | 5.5.5 | 8.8.8 | | " " left " |
| 9.20 | 5.4.5 | 8.8.8 | | |
| 10 | 2.2.2 | 4.5.4 | | |
| 11 | 2.1.2 | 3.4.3 | | Very weak heart contractions. The diastoles are of very different duration. The auricles are over-filled with blood. |

*Local Action on Nerve-trunks.*—Cocaine in weak solution (1 per cent.), when applied directly to nerve-trunks, after a very transient period of increased irritability, depresses greatly, and finally paralyzes the nerve. If a strong solution is applied (4 per cent. or more) the action is almost instantaneous, and is preceded by no period of increased irritability. If, however, the solution is not left in contact with the nerve for too long a time, it will regain all of its former irritability in from 1½ to two hours.

A preparation of the sciatic nerve and gastrocnemius muscles of a frog is made in the usual manner. It is arranged (in as fresh a state as possible) on the muscle-telegraph of Prof. Dubois Raymond, and is then connected with the induction coil of his sliding induction apparatus, a simple key intervening. The nerve is placed in a moist chamber, and non-polarized electrodes are used. After determining the irritability of the nerve a solution of cocaine (1 per cent.) is applied by means of a camel's hair pencil. The figures in the second column of the table indicate the distance in centimetres that the induction coil is from the primary, where the contraction of the muscles takes place.

### EXPERIMENT XII.

| Time. | Centimetres. | Amount Cocaine. | Remarks. |
|---|---|---|---|
| 10.10 | 70 | 1 per cent. | |
| 10.20 | 43 | | |
| 10.25 | 37 | 1 per cent. | |
| 10.30 | 33 | | Contractions very feeble, cannot produce tetanus. |
| 10.35 | No response | | |

### (CONTROL)—EXPERIMENT XIII.

Conditions same as in XII. Water used first, then Cocaine.

| Time. | Centimetres | Water and Sol. Cocaine. | Remarks. |
|---|---|---|---|
| 10.15 | 93 | Water | |
| 10.20 | 97* | | 97 is the limit of the distance possible between the two coils. |
| 10.30 | 97 | Water | |
| 10.40 | 97 | | |
| 10.45 | 97 | Cocaine Sol. 1 per cent. | Active Contractions. |
| 11.15 | No response | | The irritability rapidly diminished after the application of cocaine in solution, and the nerve was completely paralyzed at the end of thirty minutes. |

* The irritability of a nerve is often greater soon after removal, than at the time of removal

### EXPERIMENT XIV.

Conditions same as XIII.

| Time. | Centimetres. | Water or Sol. Cocaine. | Remarks. |
|---|---|---|---|
| 11.10 | 87 | Water | |
| 11.20 | 97 | | |
| 11.40 | 97 | Water | Very active. |
| 11.50 | 46 | | |
| 12.05 | 42 | | After having been in water for one hour and twenty-five minutes, all irritability disappeared in six minutes when sol. cocaine applied. |
| 12.35 | 36 | Cocaine Sol. 1 per cent. | |
| 12.41 | No response | | |

EXPERIMENT XV.

Cocaine in 4 per cent. Solution used.

| Time. | Centimetres. | Sol. Cocaine 4 Per Cent. | Remarks. |
|---|---|---|---|
| 10.50 | 50 | | |
| 10.55 | 50 | 4 per cent. | |
| 10.56 | 47 | | |
| 10.59 | No response to the strongest current. | | |

In a number of other experiments with the action of a 1 per cent. solution of cocaine on nerves, all irritability had disappeared in from fifteen to thirty-five minutes.

EXPERIMENT XV.

Medulla of frog destroyed ; sciatic nerve of one leg carefully exposed, as little injury being done to the vessels as possible; the irritability of the nerve is accurately determined with the sliding induction apparatus. A solution of cocaine is now applied until the nerve only responds to the stimulus. A very strong current—normal salt solution (3-4 per cent.)—is then substituted for it, and in the course of an hour and a half the irritability of the nerve has completely, or almost completely, returned.

EXPERIMENT XVI.

Medulla of the frog destroyed. Sciatic nerve exposed, and a cord is passed carefully under it so as to include all of the limb, excepting the nerve, and is firmly tied; frog is poisoned by a moderate dose of cocaine. Then the irritability of the nerve and muscles of the leg not so shut off from the insulation is tested as compared with that of the bound leg, and is found to be much less. If the irritability is again tested after the lapse of an hour and a half (if the amount of the poison given has not been too large), it is found that it has become as great in the poisoned leg as in the other. From these two experiments it is seen that the irritability of a nerve, whether diminished by the local action of the cocaine or by cocaine in the circulation, regains its lost irritability in about the same length of time. It would, therefore, seem as if the action of the drug on the nerve was in both cases merely a local one.

EXPERIMENT XVII.

If the nerve from a nerve-muscle preparation, prepared in the usual manner, be immersed for a few seconds in a solution of cocaine (1 per cent.) and then be removed, its irritability is much increased. The slightest touch of a steel instrument will throw the muscles into violent tetanic contractions. If it be now immersed for a longer time the excitability becomes much diminished and is finally lost to all forms of stimulation. When the nerve is first placed in the solution no irritation is caused by the immersion.

EXPERIMENT XVIII.

If a nerve-muscle preparation be made from a frog poisoned by cocaine, the irritability of the nerve is found to be much diminished (this is also true of the excitability of the muscle). The strength of current required to produce a muscular contraction is much greater than for that from a normal frog. The muscular pulsations are shorter and less powerful, and it is impossible to produce tetanic contractions if the amount of the poison given was at all large. When the interrupted current is passed through it, for some time several short, quick, feeble pulsations occur, and then it responds no more to the irritation. The action is very similar to that produced by stimulation applied to a nerve poisoned by the local application of a solution of cocaine.

*Influence on the Power of Co-ordination.*—The influence of cocaine on the nervous system would not be complete without an allusion to the apparently marked effects of the drug on the power of co-odination, which all frogs poisoned with cocaine show. This is particularly marked after doses of moderate sizes.

*Action on Striated Muscles.*—Small doses of cocaine have little effect on the excitability of the striated muscles. But large doses diminish very decidedly their excitability. The character of the muscular contractions called forth by electric stimulation is greatly altered. The pulsations are shorter, slower and more feeble, and the excitability is soon exhausted. The local action of cocaine on muscular tissues is very similar in every way to that found in general poisoning.

### EXPERIMENT XIX.

Gastrocnemius of frog (prepared in the usual manner without the nerve) is placed in the Dubois Reymond Muscle Telegraph Apparatus, and is then connected with the induction coil of his sliding Induction Apparatus.

(The numbers in the second column as before, represent the number of centimetres that the induction coil is distant from the primary coil when contraction occurred.)

| Time. | Centimetres. | Amount Cocaine. | Remarks. |
|---|---|---|---|
| 12.03 | 24 | | |
| 12.05 | 23 | 1 per cent. | |
| 12.07 | 18 | | |
| 12.08 | 17 | | |
| 12.10 | 17 | 1 per cent. | |
| 12.11 | 16 | | |
| 12.18 | 16 | 1 per cent. | |
| 12.22 | 15 | | |
| 12.26 | 13 | | Responds feebly and after two or three contractions responds no more. |
| 12.30 | 12 | | |
| 12.40 | Very feeble response to a very strong current. | | |

### EXPERIMENT XX.

Preparations and conditions same as in XIX. Sol. Cocaine 2 per cent.

| Time. | Centimetres. | Amount Cocaine. | Remarks. |
|---|---|---|---|
| 12.35 | 26 | | |
| 12.35 | 26 | 2 per cent. | |
| 12.38 | 20 | | |
| 12.40 | 18 | | Quick short pulsations. |
| 12.44 | | 2 per cent. | " " " |
| 12.50 | 16 | 2 per cent. | |
| 12.55 | 15 | | Contractions slow and feeble. |
| 1.07 | 12 | | Responds very feebly. |
| 1.15 | 5 | | |
| 1.25 | No response | | |

(Cocaine applied by means of a camel's hair pencil.)

The application of cocaine in substance to a muscle causes a gradual diminution in the excitability, until finally, after from twenty to twenty-five minutes, there is no response to the strongest interrupted current. It requires many hours for a muscle to regain its excitability when it is to any considerable extent destroyed by the local action of cocaine.

The action of cocaine on the frog may thus be summed up as follows :

1. It has a powerful local anæsthetic action on the skin, mu, cous membrane and eye. It usually produces mydriasis.

2. It has a depressant action on the heart, reduces first the force and frequency of its pulsations, and finally paralyzes it (first the ventricles and then the auricles) in diastole.

3. In small doses it at first slightly increases the number of the respirations, then decreases them ; and in large doses- diminishes the number from the first, and finally causes death from paralysis of respiration.

4. It at first slightly heightens and then depresses the reflex action of the spinal cord.

5. In small doses, it at first slightly increases the irritability of the sensory nerves, then depresses their irritability, and in large doses, depresses from the first.

6. It has a depressant action on the motor nerves in both large and small doses (not very small).

7. It paralyzes the pneumogastrics.

8. The moderate-sized doses diminishes the excitability of the striated muscular fibre.

9. From a comparison of the local and constitutional action on the different organs and tissues, it is rendered probable, at least, that its constitutional action is wholly a local one, exercised alike on all parts, for which it has a chemical affinity, through its presence in the blood.

The writer hopes, not far in the future, to be able to report in the same manner the local and general action of cocaine on the organs and tissues of the warm-blooded animals.

